# Cryptosexuality and the Genetic Diversity Paradox in Coffee Rust, *Hemileia vastatrix*

**DOI:** 10.1371/journal.pone.0026387

**Published:** 2011-11-15

**Authors:** Carlos Roberto Carvalho, Ronaldo C. Fernandes, Guilherme Mendes Almeida Carvalho, Robert W. Barreto, Harry C. Evans

**Affiliations:** 1 Departamento de Biologia Geral, Universidade Federal de Viçosa, Minas Gerais, Brazil; 2 Departamento de Fitopatologia, Universidade Federal de Viçosa, Minas Gerais, Brazil; 3 CAB International, Egham, Surrey, United Kingdom; University of Minnesota, United States of America

## Abstract

**Background:**

Despite the fact that coffee rust was first investigated scientifically more than a century ago, and that the disease is one of the major constraints to coffee production - constantly changing the socio-economic and historical landscape of the crop - critical aspects of the life cycle of the pathogen, *Hemileia vastatrix*, remain unclear. The asexual urediniospores are regarded as the only functional propagule: theoretically, making *H. vastatrix* a clonal species. However, the well-documented emergence of new rust pathotypes and the breakdown in genetic resistance of coffee cultivars, present a paradox.

**Methods and Results:**

Here, using computer-assisted DNA image cytometry, following a modified nuclear stoichiometric staining technique with Feulgen, we show that meiosis occurs within the urediniospores. Stages of spore development were categorised based on morphology, from the spore-mother cell through to the germinating spore, and the relative nuclear DNA content was quantified statistically at each stage.

**Conclusions:**

Hidden sexual reproduction disguised within the asexual spore (cryptosexuality) could explain why new physiological races have arisen so often and so quickly in *Hemileia vastatrix*. This could have considerable implications for coffee breeding strategies and may be a common event in rust fungi, especially in related genera occupying the same basal phylogenetic lineages.

## Introduction

**Figure 1:**
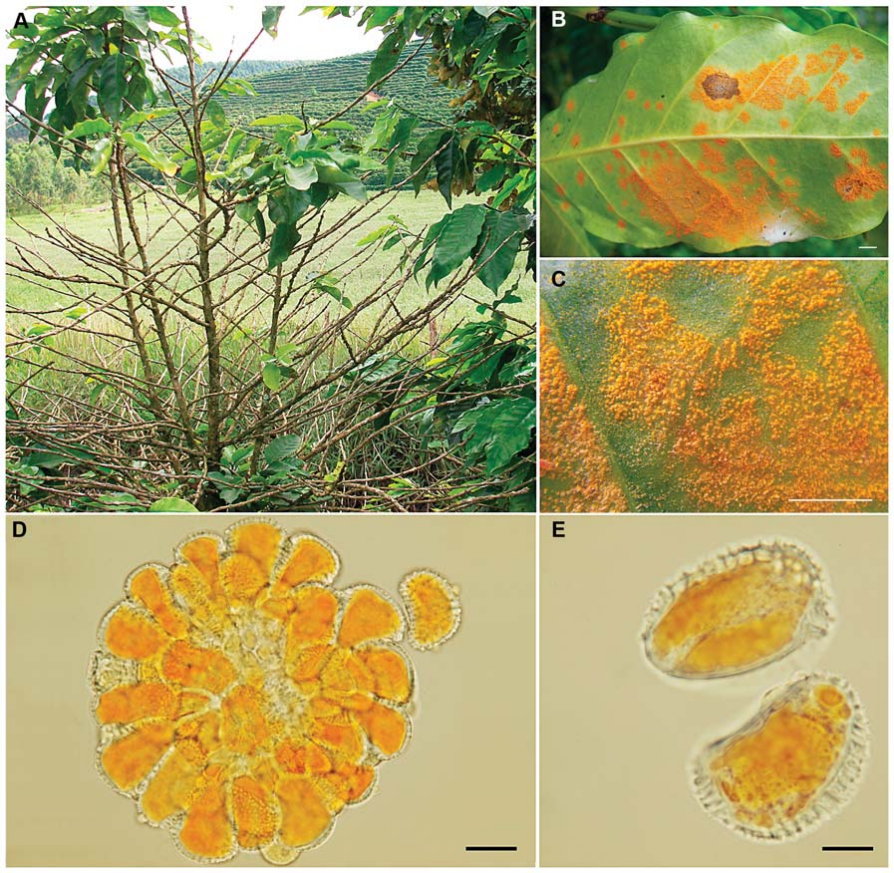
Symptoms and morphology of coffee leaf rust, *Hemileia vastatrix*. (**A**) Defoliation in a coffee plantation, Coimbra, Minas Gerais, Brazil; (**B**) Leaf symptoms on abaxial surface (bar = 0.5 cm); (**C**) Detail of suprastomatal uredinial pustules coalescing over lower leaf surface (bar = 0.5 cm); (**D**) Uredinium showing arrangement of spores (bar = 20 µm); (**E**) Urediniospores - showing the thickened, heavily-ornamented or verrucose upper wall – containing carotenoid lipid guttules imparting the yellow-orange colour (bar = 10 µm).

Coffee, after petroleum, is the world's most heavily traded commodity [1]. *Hemileia vastatrix* Berk. & Broome, the causal agent of coffee leaf rust, was first described by the Victorian mycologist M.J. Berkeley [2], based on specimens sent from Ceylon (now Sri Lanka), where it was causing serious problems in the burgeoning and highly profitable coffee plantations. Subsequently, disease pressure led to the abandonment of coffee cultivation in that country in favour of tea [3]. Since then, the rust has been changing the socio-economic and historical landscape of this crop throughout the Tropics [4]. Essentially, the modern view of the rust life cycle [5] is no different from that proposed by the pioneering plant pathologist H.M. Ward, working in Ceylon more than a century ago [6], [7]. The generic name *Hemileia*, reflects the characteristic half-smooth (half-rough) morphology of the supposed asexual spores or urediniospores, which function as the dominant dispersal and infective propagules (Fig. 1E). The role of the sexual spores or basidiospores - produced during germination of the ephemeral teliospores *in situ* on the coffee leaf - remains uncertain, except that they “do not infect coffee, but no alternate host is known yet” [5]. This is despite repeated attempts over the years to infect coffee plants with these spores [5], [8], [9], [10], [11], [12]. Thus, the prevailing but unproven hypothesis is that this rust species is heteroecious [13] and that a secondary or haplophase host, purportedly an orchid, exists in the centre of origin or diversity of the genus *Coffea* in East-Central Africa [14], [15].

Genetic abnormalities in the urediniospores were first noted by Rajendren [16], [17], who described the occurrence of karyogamy and meiosis within these supposed asexual spores, based on cytological evidence. He termed these “uredinioid teliospores”, but, subsequently, this nuclear process was disputed [18], and it has been ignored since, within the context of coffee rust research. However, a similar nuclear behaviour in the urediniospores of a related rust fungus, *Maravalia cryptostegiae* (Cummins) Y. Ono, has also been reported more recently [19]. Following molecular studies, these two rust species have now been shown to share a common ancestry, and both the genera *Hemileia* and *Maravalia* occupy a basal position within the *Pucciniales* and thus represent the oldest lineages [20], [21]. Therefore, contrary to previous hypotheses –especially the prevailing one, positing that primitive rusts occur only on primitive host plants [22] - this would be reflected in a simple, unexpanded or only partially expanded autoecious life-cycle, whilst heteroecism is a more advanced condition which never developed in these primitive tropical rusts [23].

If this hypothesis is correct, then the basidiospores should infect the coffee host. Thus, the failure to achieve infection remains a stumbling block to understanding the life cycle and, most importantly, how new races or pathotypes of the rust arise to overcome host resistance: a recurring theme since it was first documented from India in the 1930s [24]. There are indications, however, that development and germination of the teliospores is also abnormal with asynchronous nuclear division, resulting in basidiospores with significant variation in shape, size and chromatin content [25], [26], [27] - an analogous situation to that reported for *M. cryptostegiae* based on cytological evidence - which led to the alternative hypothesis that these represent primitive autoecious rusts in which the sexual spores are vestigial and non-functional, and that the urediniospores have multiple functions: sexual recombination; dispersal; infection and survival [19]. Nevertheless, given the high profile of coffee rust, more convincing evidence is needed to support this hypothetical life cycle. Using more advanced techniques, we set out to test the theory that sexual reproduction occurs within the urediniospores and that this is the dominant event in the life cycle of coffee rust and thus the source of the paradoxically high genetic diversity of this critically important plant pathogen.

## Results and Discussion

**Figure 2:**
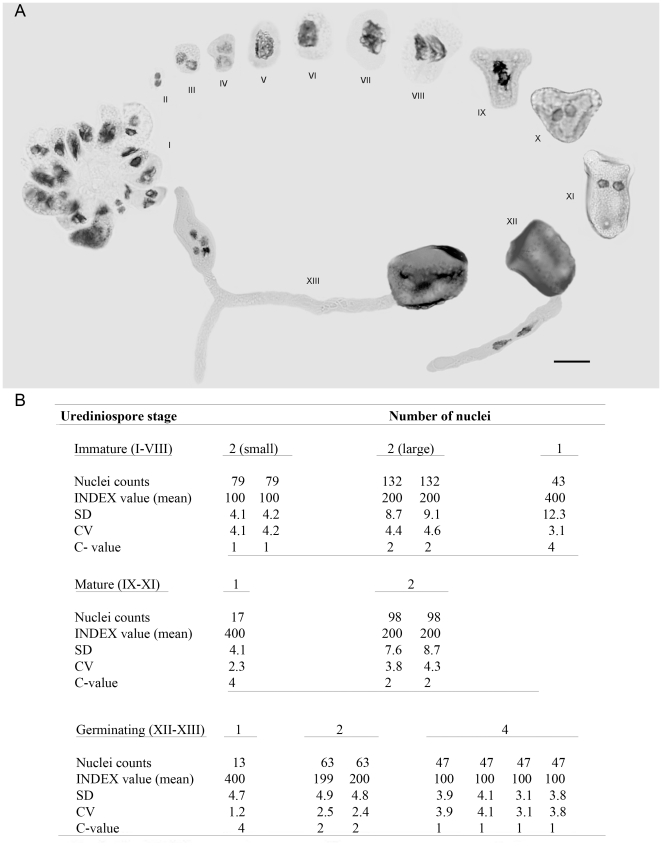
Nuclear cycle observed within urediniospores of *Hemileia vastatrix* as revealed by image cytometry with quantification of DNA content after Feulgen staining. (**A**) Sequence of nuclear events from spore-mother cells in uredinium (I) through karyogamy and meiosis I as the spores develop, reach maturity and enter interphase (XI), followed by a delayed meiosis II which occurs when the spores germínate to form appressoria (XII–XIII; bar = 10 µm); (**B**) Table of nuclear C-values relating to the stage of spore development shown in **A**: immature cells with 2 small nuclei (II), 2 large nuclei (III, IV) and 1 large nucleus (V, VI); maturing spores with 2 nuclei following first meiotic division (X, XI); finally, the germ tube with 2 (XII) and then 4 nuclei (XIII).

**Figure 3:**
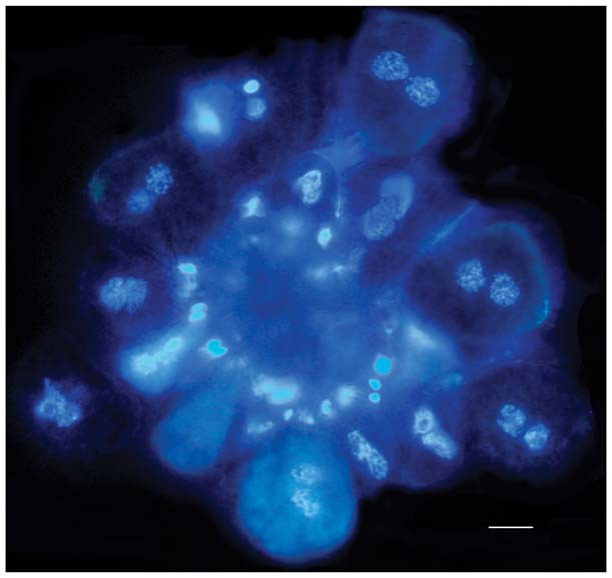
Uredinium of *Hemileia vastatrix* stained with DAPI. Series of nuclear events in the spore-mother cells and immature urediniospores (I–VII), prior to meiosis (bar = 10 µm).

**Figure 4:**
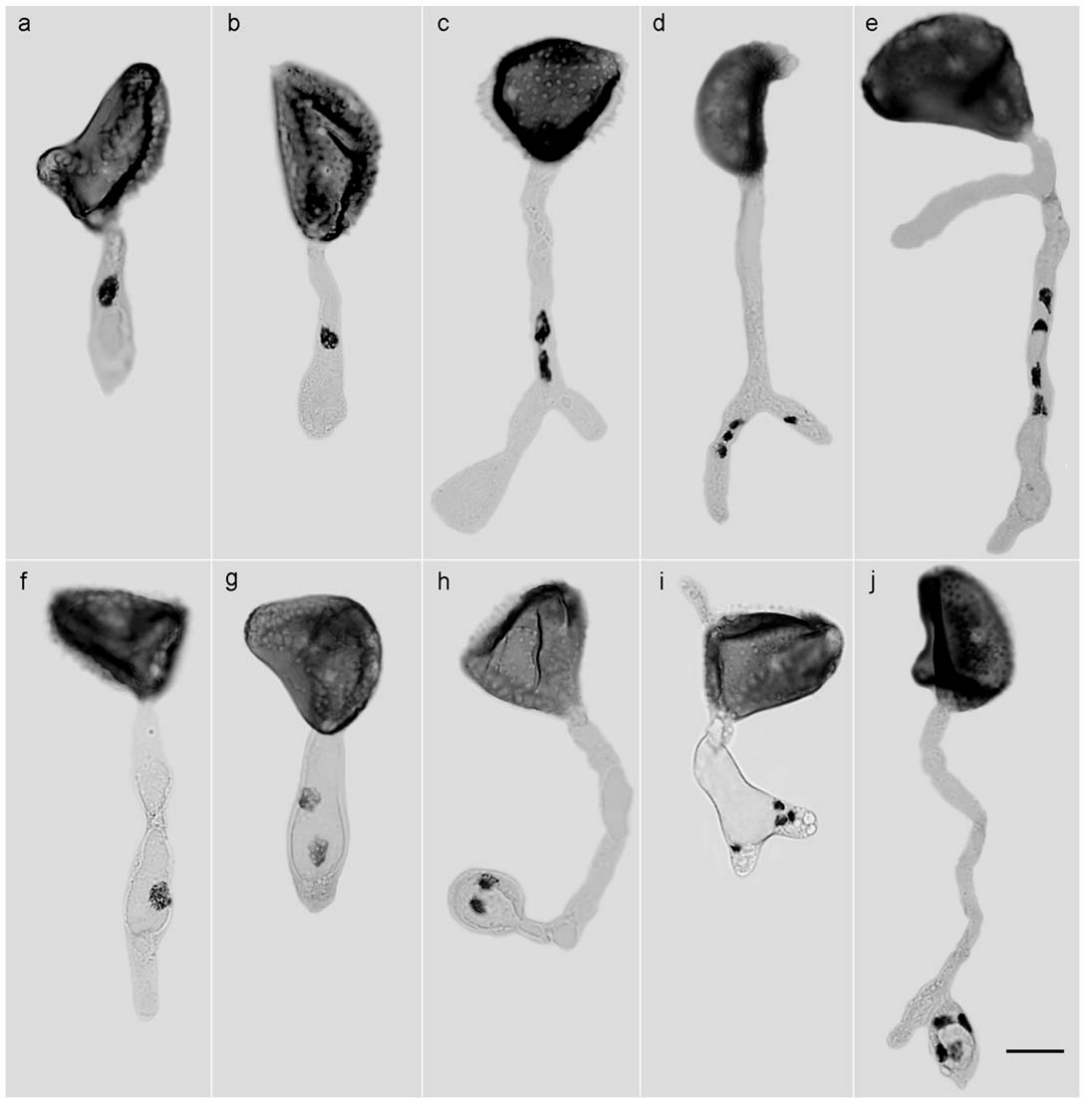
Events in germinating urediniospores of *Hemileia vastatrix* containing a single nucleus. (**a, b, f**) G_2_ diploid nucleus (C-value = 4, see Fig. 2B) moving from spore into the germ tube; (**c, g, h**) Two nuclei (C-value = 2) after meiosis I occurs as germ tube divides or prior to appressorial formation; (**d, e**) Four nuclei (C-value = 1) after meiosis II in the germ tube or (**i, j**) appressorium (bar = 10 µm).

After nuclear stoichiometric staining, urediniospores in all stages of development, including those conditioned to germinate, were examined using DNA image cytometry. Since *H. vastatrix* is a biotroph, it was not possible to follow the development of urediniospores using a time-lapse approach. Instead, spore developmental stages were categorised based on morphology. The most common sequence in the non-germinated spores is shown in Figure 2A: from the spore-mother cell in the young uredinium (I), with clustered, smooth, thin-walled spores showing a progressive centrifugal size increase (I–VIII), through to the loose, significantly bigger mature urediniospores developing thick, heavily-ornamented upper walls (IX–XI); and, finally, germinated urediniospores showing the formation of the pre-infection structures (XII–XIII, germ tube and appressorium). The relative nuclear DNA content (C-value) was quantified statistically at each stage of development and this is expressed as mean index value (INDEX) (Fig. 2B). The spore-mother cell in the uredinium (I) and the newly released, immature urediniospore (II), with an ill-defined spore wall, contain two small nuclei each with an INDEX of 100 (C-value = 1). As the spores increase in size and the nuclei enlarge, the DNA content doubles (III, IV), each nucleus showing an INDEX of 200 (C-value = 2). These series of events within a uredinium were also captured with DAPI staining (Fig. 3). This occurs prior to karyogamy when the two G_2_ haploid nuclei fuse to form the G2 diploid urediniospore – which is still immature as signified by the thin, non-ornamented wall (V, VI) - with an INDEX of 400 (C-value = 4). The spores undergo meiosis I, with clear evidence of prophase (V, VI), early anaphase (VII), anaphase (VIII) and late anaphase (IX), shown by the clustering of chromosomes on the spindle apparatus and their transition to the poles. As the urediniospore develops, the thicker heavily-ornamented spore wall is laid down; eventually maturing with two G_2_ haploid nuclei (X, XI), each with an INDEX of 200 (C-value = 2). However, a minority of urediniospores (<2%) reach maturity without undergoing meiosis, as evidenced by the INDEX 400 (C-value = 4) (data not shown). When the urediniospores are stimulated to germinate, the greater majority show the two nuclei streaming into the germ tube (XII), and the beginning of meiosis II which culminates in four haploid nuclei each with an INDEX of 100 (C-value = 1), usually found within a swollen, appressorium-like structure (XIII). The less common event of delayed meiosis - with a single diploid nucleus (C-value = 4) entering the germ tube, followed by meiosis I and II, either in the elongating tube or in the appressorium - is shown in Fig. 4.

Our results, which are based on quantitative data through measurement of DNA content, confirm those reported much earlier, based on a qualitative interpretation of nuclear events using standard cytology [16], [17]. The data obtained from this DNA image cytometry study provide compelling evidence that meiosis occurs in the urediniospores. However, it could also be argued that some of the immature spores are, in fact, teliospores: a highly unlikely event since teliospores are extremely rare, particularly during the period when the samples were collected [27]. In addition, there is evidence of asynchronous nuclear division in urediniospores of *H. vastatrix* resulting in variable chromatin content, but this is not common [23], and in our categorisation of nuclear events, only the most representative sequences were chosen.

This hidden sexual reproduction disguised within the asexual (anamorph) stage, or cryptosexuality, could explain why new physiological races of the rust have arisen so quickly in this supposed clonal rust species to overcome the host resistance developed by plant breeders – firstly, in India [24] and, more recently, in Brazil [28], [29] and Colombia [30]. The latter study concluded that *H. vastatrix* has a high mutation rate, based on an analysis of the ITS1 and ITS2 regions.

We can now begin to understand the reasons for this high genetic diversity and so improve future breeding programmes to ensure that resistance is more durable. This may also provide insights into the on-going evolution of *H. vastatrix* and related primitive rust species; such as *M. cryptostegiae*, which is playing a pivotal role as a classical biocontrol agent for management of the invasive alien rubber-vine weed, *Cryptostegia grandiflora* (Roxb.) R. Br. (Asclepiadaceae), in Australia [31]. For both species, this is reflected in abnormal germination and asynchronous nuclear events not only in the true teliospores [18], [19], [25], [27], but also in the urediniospores on rare occasions [19], [23], [32]. It has now been shown that the main coffee species of commerce, *Coffea arabica* L., is an allotetraploid of recent hybrid origin, with two East African lowland forest species - *C. canephora* Pierre ex A. Froehner and *C. eugenioides* S. Moore - as the progenitors [33]. It is postulated here that, as this new hybrid species adapted to the more open and drier upland habitats of north-west Kenya and south-east Ethiopia, *H. vastatrix* has modified its life cycle to marginalize the ephemeral, thin-walled teliospores in favour of the more robust and now multifunctional urediniospores. This is the rust ‘variant’ that has moved around the world with its *C. arabica* host [4]. It is further proposed that, in the absence of selection pressures, the life cycle of *Hemileia vastatrix* on the diploid forest progenitors has remained unchanged and, therefore, that the sexual teliospores are still fully functional, whilst the urediniospores serve only as repetitive, dispersal propagules in these wetter forest habitats.

The genetic plasticity and cryptosexuality of coffee rust may be common to other rust fungi, notably those which occupy the same basal phylogenetic lineages [20], [21], but may also occur throughout the *Pucciniales*, providing a far reaching explanation for similar paradoxically high genetic diversities for supposedly clonal species.

In conclusion, cryptosexuality in *Hemileia vastatrix* could have considerable implications not only for future coffee breeding strategies, but also for sustainable long-term coffee production, especially since global warming could increase the range and thus the economic impact of coffee rust in countries such as Brazil [34].

## Materials and Methods

### Ethics statement

No specific permits were required for the described field studies. All field sites visited were on property belonging to the Universidade Federal de Viçosa (UFV), and no endangered or protected species were involved in the studies.

### Rust material

Coffee leaves, infected naturally in the field by *H. vastatrix*, were collected from experimental sites and organic coffee farms in the immediate vicinity of Coimbra (Fig. 1A) and Viçosa, Minas Gerais, Brazil, between January 2008 and March 2010.

### Slide preparation

Urediniospores were collected in sterile plastic tubes by gently brushing the rust pustules (uredinia) present on the abaxial surface of leaves (Fig. 1B–D) with a soft paint brush. Leaves were inspected beforehand using a stereoscopic microscope to select only those free of mycophagous arthropods - especially mites and dipteran larvae - and mycoparasites. Spores (Fig. 1E) were sprinkled onto one half of sterile glass microscope slides, previously kept in a refrigerator at 5°C for 5 min to promote condensation and simulate dew formation. The slides were then transferred to a humid chamber and maintained in an incubator in the dark at 25°C for 24 h to germinate. Previous studies [12] had shown that under these conditions urediniospores germinate to produce short, stout germ tubes; often swollen apically or laterally in the form of an infection pad or appressorium. After removal from the germination chamber, dry urediniospores were dusted onto the other half of the slides, to have a standardized comparison between germinated and non-germinated spores. The slides were air-dried and placed on a hot plate (surface temperature 50°C) for 10 min. To preserve the integrity of the chromatin, the slides were fixed in 70% ethanol for 24 h at −20°C, then air-dried and a drop of 45% acetic acid was placed centrally on each slide. By using a cover slip (50×22 mm), firm pressure was applied to the material to separate the protoplasm from the spore wall. This greatly facilitated observation of the nuclei, especially in the thick-walled, mature urediniospores. Removal of the coverslips was made easier after transferring the slides to a refrigerator at 5°C for 5 min.

### Feulgen reaction

The following method, modified from Mendoça *et al.* [35], was used. The slides were placed in a fixative solution of methanol: 37% formaldehyde: acetic acid (Merck®), 17∶5∶1, for 12 h at 25°C, washed in distilled water, air-dried, and hydrolyzed in 5 M HCl (Merck®) for 18 min at 30°C. After hydrolysis, slides were stained with Schiff's reagent (Merck®) for 12 h at 4°C. Finally, slides were washed twice for 1 min in 0.5% SO_2_ aqueous solution (Merck®), then in distilled water for 30 seconds, and mounted in immersion oil (ND = 1.525) (Carl Zeiss®).

### Microscope instrumentation and image analysis system

Image Cytometry (ICM) analysis was carried out at the Laboratório de Citogenética e Citometria, Universidade Federal de Viçosa (UFV). The images were captured with a monochromatic charge-coupled device (CCD) digital video camera of 12 bits gray and a frame grabber card (Photometrics CoolSNAP Pro®). This camera was assembled on a trinocular photomicroscope (OlympusTM BX-60) equipped with: (a) source of stabilized light, (b) PlanApo ×60 oil immersion objective with 1.40 numeric aperture, (c) Aplanat Achromat condenser with aperture 1.4 and (d) neutral density filter (ND6). The video camera was further coupled to a Pentium 4 HT computer (Dell® Optiplex GX 620) with the Image Pro® - Plus 6.1 software (Media Cybernetics®).

### Calibration and set-up of the image analysis system

The microscope was adjusted prior to each nuclear image capture session by the Köhler method for the optimal light path to reduce stray light and the image cytometry set-up followed previously standardized procedures [36], [37]. Spatial calibration was performed using a slide micrometer scale (OlympusTM) and tools from the Image Pro®-Plus 6.1 (Media Cybernetics®) analysis system. This procedure established the conversion unit from pixel to micrometer. Calibration and evaluation of the image analysis system were performed and consisted of three tests: (i) stability; (ii) linearity, accomplished with a set of certified neutral density filters: 0.15, 0.30, 0.40, 0.60, 0.90 and 2.50 (Edmund Industrial Optics®), and; (iii) uniformity, using 11 stepped density filters (Edmund Industrial Optics®). For each individual slide, the nuclear capture session was accomplished using the optical density (OD) range calibration, employing the microscope light intensity control and the histogram live-window (gray scale) of the Image Pro® - Plus 6.1 analysis system. The diaphragm iris was closed to a slightly larger size than the image size. OD range was determined by opening the software histogram live-window and adjusting the microscope light intensity control. The highest gray level (peak moves to right) was slightly lower than maximum value on the gray scale.

### Experimental design for C-value determination

General nuclear frames of germinating urediniospores (in various stages of development) were captured, as well as those from non-germinated spores on the other half of the slide. Image collection of each nucleus type was arranged in order to compare the integrated optical density (IOD) of samples from both halves of the slide. These 12-bit digital images were saved and the nucleus was segmented using software tools. Since the pixels do not have an intrinsic value, the spatial and optical density (OD) calibrated values were applied automatically to them by the software tool look-up table. The software algorithm automatically multiplied the nuclear area (µm^2^) by the average OD, resulting in the IOD formula, IOD = Σ^n^_1_−log10 (IFi/IBi) [37], where - IOD = nuclear IOD value; n = total number of pixels in the nucleus; IFi = intensity of the foreground (nuclear) pixel; IBi = intensity of the background (clear area) pixel. The nucleus lowest IOD value was converted to 100 INDEX value, and this corresponded to 1 C value.

### Statistical analysis

The relative nuclear genome size values were statistically analyzed. The INDEX values, obtained from all nuclei analyzed (n = 1005), were plotted using the software SPSS v 13 for Mac.

### DAPI staining

By gently brushing newly-rusted leaves, the suprastomatal uredinia were readily removed and collected on glass slides containing McIlvaine's buffer (pH 7.0), with 2.0 µ/ml DAPI (4′, 6 - diamidino -2- phenylindole, Sigma). After staining in the dark for 5 min at room temperature, slides were rinsed, air dried, mounted in the same buffer, and observed with an epifluorescent microscope fitted with a UV filter (WU cube filter, Olympus).
